# Mechanical Ventilation during Bronchiolitis: The Macklin Effect

**DOI:** 10.5334/jbsr.3410

**Published:** 2024-03-11

**Authors:** Badri Haider, Paolo Simoni

**Affiliations:** 1 Radiology Resident at the Queen Fabiola Children’s Hospital, Brussels, Belgium; 2 Chief of Pediatric Radiology Department of the Queen Fabiola Children’s Hospital, Brussels, Belgium

**Keywords:** Bronchiolitis, Barotrauma, Mechanical ventilation, Macklin effect

## Abstract

This is a case of barotrauma imaging (Macklin effect) after invasive mechanical ventilation in a 14-week-old newborn with complicated bronchiolitis.

*Teaching point:* Imaging could help us improve defining the anatomical boundaries of the Macklin effect, an incompletely known anatomo-physiological entity.

## Introduction

Bronchiolitis is one of the most common diseases in children younger than 2 years [1] and leaves usually no sequelae. Its diagnosis remains clinical and imaging is unnecessary in uncomplicated bronchiolitis [2]. Mechanical ventilation is indicated in case of clinical worsening and can cause barotrauma, especially in case of pulmonary infection [3]. Leaking of air can cleave or occupy virtual spaces in the mediastinum and cause highly specific lesions [4]. This is a case of barotrauma imaging after invasive mechanical ventilation in a newborn child with complicated bronchiolitis.

## Case Report

We report a case of a 4-month-old girl delivered at 32 weeks and 4 days. A C-section was chosen due to hypoxia with low Apgar scores, treated with continuous positive airway pressure and a high-pressure nasal cannula until day 38 of life.

**Figure 1:**
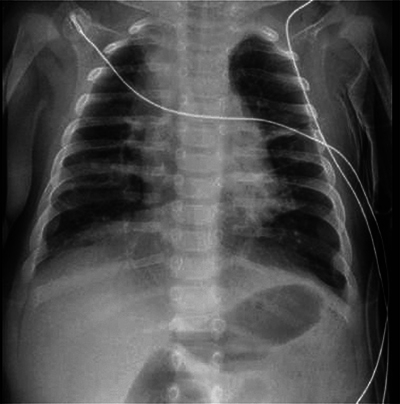
Chest antero-posterior radiograph. Multiple pulmonary opacities and an air bronchogram suggesting pneumonia.

**Figure 2:**
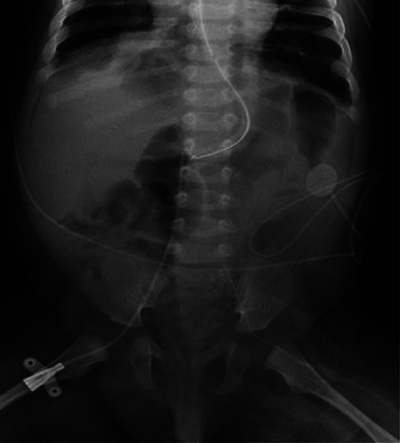
Abdominal radiograph. Hyperinflation and lateral abdominal subcutaneous emphysema.

At 14 weeks of corrected age, the patient developed an episode of bronchiolitis. Thoracic radiography showed multiple pulmonary opacities and an air bronchogram suggesting pneumonia (Figure 1), treated with antibiotics, and abdominal radiography showed hyperinflation (Figure 2).

**Figure 3:**
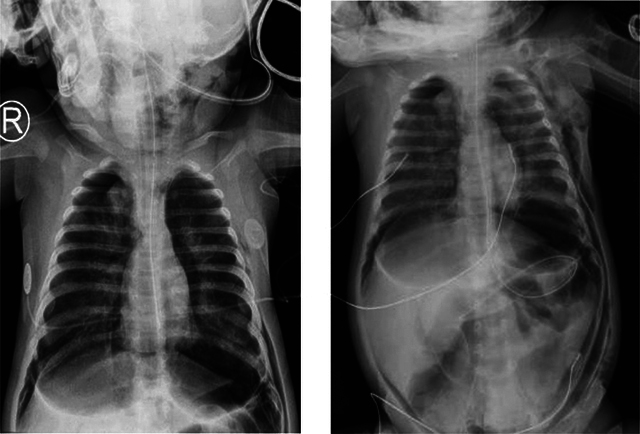
(A, B) Chest and abdominal antero-posterior radiograph. Free air (subcutaneous emphysema) located at the infra-thoracic part of the chest radiograph.

Mechanical ventilation was performed to improve the patients episodes of apneas and desaturation but resulted shortly after in free air located at the infra-thoracic part of the chest radiograph (Figure 3A and B), treated by the placement of two left thoracic drains.

After two episodes of cardiac arrests, the infant was placed on a veno-venous ECMO.

**Figure 4:**
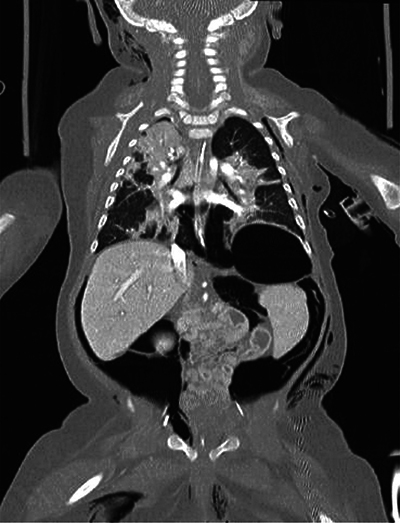
Whole body low-dose CT scanner. Latero-thoracic and right-cranial subcutaneous emphysema along with massive free air (e.g., pneumomediastinum and pneumopericardium).

**Figure 5:**
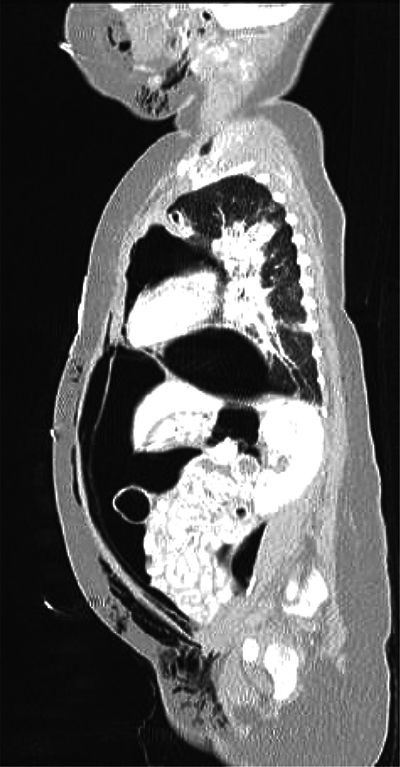
Whole body low-dose CT scanner. Latero-thoracic and right-cranial subcutaneous emphysema along with massive free air.

A whole body low-dose computed tomography (CT) scanner showed a tension pneumothorax leading to decreased cardiac output. It also showed extensive deep tissue emphysema (latero-thoracic and right-cranial), pneumomediastinum, pneumopericardium, and important retroperitoneal free air without pneumoperitoneum (Figures 4 and 5).

**Figure 6:**
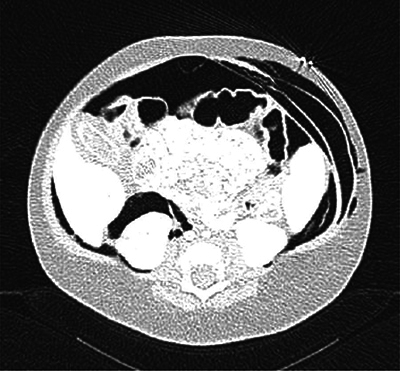
Whole body low-dose CT scanner. Cleavage of all abdominal layers produced by the free air.

Further, it showed abdominopelvic ascites, periportal, and perivascular edema. There was cleavage of all abdominal layers produced by the free air (Figure 6).

**Figure 7:**
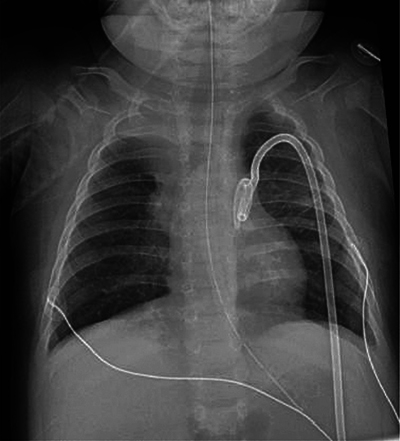
Chest radiograph. Total recovery of the free air throughout the body due to drainage and tissue resorption.

Clinicians decided to decrease pressure ventilation and drain the left pneumothorax, resulting in total recovery after two weeks due to drainage and tissue resorption (Figure 7).

Three months later, the patient deceased on cardiopulmonary arrest after an episode of fever and shock. Anatomo-pathological etiological evidence of death was not performed due to the delay between the patient’s death and the autopsy proposal.

## Discussion

Barotrauma is the presence of extra-alveolar air during mechanical ventilation, including subcutaneous emphysema, pneumomediastinum, pneumopericardium, and pneumoperitoneum [5,6]. The presence of a pneumomediastinum within our patient ultimately caused compressive heart failure and cardiac arrests.

During the first years of life, the incidence of bronchiolitis is around 11% to 15% [5]. Depending on the severity of the infection, there are at least five hospitalizations for every 1,000 children younger than 2 years of age [7].

Barotrauma frequency following invasive mechanical ventilation is rare and estimated at 4% to 15% [8,9].

Medical imaging techniques allowed us to quantify the amount of free air and to follow the extension of ‘free’ air throughout the body explained by the Macklin effect.

The Macklin effect is caused by a three-step pathophysiologic progress when blunt traumatic alveolar rupture causes dissection along broncho-vascular sheaths and spreading interstitial emphysema, causing pneumomediastinum, and ultimately progressing throughout the different body interstitia. The first imaging choice is CT, which shows interstitial emphysema adhering to a bronchus and pulmonary blood vessel [10].

The interstitium/interstitial space is the primary source of lymph and a major body fluid compartment supported by collagen fibers. It is found in many organs and seem to be connected to each other without interruption. Recent research considers the interstitium as an organ (a tissue group uniquely structured performing a specialized task). The interstitium task is hydro-electrolytic and proteic homeostasis, but also morphogenesis, cell migration, and cell–cell signaling. This is especially important for immune regulation, requiring continuous cell–cell, antigen, and cytokine communication [11].

The composition, biophysical, and chemical properties of the interstitium vary for each organ and its (patho)physiological state [12]. Its global volume is about 15% to 16% of body weight, varying in abundance per organ [13].

Since excessive interstitial fluid is reabsorbed into the lymphatics, it is not visible on chest radiographs, becoming visible in case of space-occupying lesions causing impairment in the lymphatic clearing mechanism. This causes distension of the interstitium, as seen in our case.

Oncologists specialized in metastatic processes especially have been interested in this not well-known topic [14] since interstitial anatomical boundaries are still not totally defined. Modern imaging techniques could ultimately contribute in understanding the interstitium, and it could be used when studying human body composition.
